# Modeling type 2 diabetes in rats by administering tacrolimus

**DOI:** 10.1080/19382014.2022.2051991

**Published:** 2022-03-29

**Authors:** JC Quintana-Pérez, F García-Dolores, AS Valdez-Guerrero, D Alemán-González-Duhart, MG Arellano-Mendoza, S Rojas Hernández, IM Olivares-Corichi, JR García Sánchez, JG Trujillo Ferrara, F Tamay-Cach

**Affiliations:** a Laboratorio de Investigación en Bioquímica Aplicada, Sección de Estudios de Posgrado e Investigación y Departamento de Formación Básica Disciplinaria, Escuela Superior de Medicina, Instituto Politécnico Nacional, Plan de San Luis y Díaz Mirón, Casco de Santo Tomas,Miguel Hidalgo, Ciudad de México, México; b Departamento de Patología, Instituto de Servicios Periciales y Ciencias Forenses, Tribunal Superior de Justicia de la Ciudad de México, Ciudad de México, México; c Laboratorio de Investigación en Enfermedades Crónico Degenerativas, Sección de Estudios de Posgrado e Investigación, Escuela Superior de Medicina, Instituto Politécnico Nacional, Plan de San Luis y Díaz Mirón, Casco de Santo Tomas, Miguel Hidalgo, Ciudad de México, México; d Laboratorio de Inmunología Celular y Molecular, Sección de Estudios de Posgrado e Investigación, Escuela Superior de Medicina, Instituto Politécnico Nacional, Plan de San Luis y Díaz Mirón, Casco de Santo Tomas, Miguel Hidalgo, Ciudad de México, México; e Laboratorio de Estrés Oxidativo, Sección de Estudios de Posgrado e Investigación, Escuela Superior de Medicina, Instituto Politécnico Nacional, Plan de San Luis y Díaz Mirón, Casco de Santo Tomas, Ciudad de México, México

**Keywords:** Wistar rat, tacrolimus, pancreatic damage, hyperglycemia, modeling type 2 diabetes

## Abstract

The prevalence of diabetes is rapidly increasing. The current number of diagnosed cases is **~**422 million, expected to reach **~**640 million by 2040. Type 2 diabetes, which constitutes **~**95% of the cases, is characterized by insulin resistance and a progressive loss of β-cell function. Despite intense research efforts, no treatments are yet able to cure the disease or halt its progression. Since all existing animal models of type 2 diabetes have serious drawbacks, one is needed that represents the complete pathogenesis, is low cost and non-obese, and can be developed relatively quickly. The aim of this study was to evaluate a low-cost, non-obese model of type 2 diabetes engendered by administering a daily high dose of tacrolimus (an immunosuppressant) to Wistar rats for 4 weeks. The biochemical and antioxidant markers were measured at basal and after the 4-week tacrolimus treatment. At week 4, the values of these parameters closely resembled those observed in human type 2 diabetes, including fasting blood glucose at 141.5 mg/dL, blood glucose greater than 200 mg/dL at 120 min of the glucose tolerance test, blood glucose at varied levels in the insulin tolerance test, and elevated levels of cholesterol and triglyceride. The tacrolimus treatment produced hypoinsulinemia and sustained hyperglycemia, probably explained by the alteration found in pancreatic β-cell function and morphology. This model should certainly be instrumental for evaluating possible type 2 diabetes treatments, and for designing new immunosuppressants that do not cause pancreatic damage, type 2 diabetes, or new-onset diabetes after transplantation (NODAT).

## 1. Introduction

Noncommunicable diseases (NCDs) were recognized by the World Health Organization (WHO) in 2016 as the leading cause of death worldwide. In 2012, NCDs constituted 71% of the 57 million registered deaths, 1.6 million of which were attributed to diabetes.^1–4^ The number of people diagnosed with this group of metabolic disorders is expected to increase from the current **~**422 million diagnosed cases to more than 640 million by 2040.^1,2,5^

Diabetes is classified into four main categories by the American Diabetes Association (ADA): gestational diabetes mellitus, type 1 diabetes, type 2 diabetes, and other specific types of diabetes triggered by particular events, such as new-onset diabetes after transplantation (NODAT).^6^ Type 2 diabetes stems from the progressive loss of β-cell function in the presence of insulin resistance.^5,6^ NODAT has not been identified with clearly defined criteria.^4,6^ It is a serious and frequent type of diabetes that occurs posterior to solid viscera transplantation, especially kidney transplantation accompanied by treatment with an immunosuppressant to avoid tissue rejection.^7–9^ Type 2 diabetes and NODAT share several risk factors and have similarities in their manifestation.

The progressive loss of pancreatic β-cell function that characterizes type 2 diabetes^5,10–12^ is associated with glucose alterations, which are found when evaluating plasma glucose after 8 h of fasting, in a random fashion, and in the glucose tolerance test. Such alterations constitute the classic symptoms of hyperglycemia or hyperglycemia crisis^4,6^ and are the basis of diagnosing the disease.

Pancreatic β cells secrete insulin in response to a high blood concentration of glucose (hyperglycemia), causing circulating glucose to enter cells. The uptake and storage of glucose are facilitated by receptors in liver, muscle, and adipose tissue.^13^ If hyperglycemia is chronic, β cells boost insulin secretion as a compensatory mechanism, but cells are eventually desensitized to the overabundance of insulin, a condition denominated insulin resistance. Hence, prolonged hyperinsulinemia and insulin resistance in peripheral tissues occur at the onset of type 2 diabetes and NODAT.^14,15^ Due to the increased demand for insulin, pancreatic β cells undergo progressive deterioration and are eventually unable to continue producing an elevated amount of the hormone. Hence, hypoinsulinemia and hyperglycemia become sustained conditions that induce the malfunction of pathways involved in glucose metabolism. These affected parameters are fundamental for the diagnosis of diabetes and any other metabolic disorder.^16–19^

Liver damage stemming from chronic hyperglycemia intensifies the activity of alanine aminotransferase (ALT) and aspartate aminotransferase (AST).^14,20^ Additionally, the polyol pathway is responsible for the elimination of approximately 30% of the glucose generated in the hyperglycemic state, leading to an increase in reductive stress.^12^

An elevated level of blood glucose provokes oxidative stress because of resulting in the excessive formation of reactive oxygen species (ROS), such as hydrogen peroxide (H_2_O_2_), nitric oxide (NO), superoxide (O_2_·^−^) and the hydroxyl radical (**^·^**OH).^14,15,21,22^ This oxidative stress causes the overactivation of poly ADP ribose polymerase (PARP) and therefore a decrease in NAD^+^.^14^ The overproduction of the superoxide anion, on the other hand, exacerbates oxidative damage by disrupting the electron transport chain. The damage is greater in complex I due to the access point of the electrons released by the oxidation of NADH.^14,15,23^ Finally, oxidation triggers apoptosis, which contributes to the destruction of pancreatic β cells and plays a central role in the pathogenesis of diabetes.^14,15,24–26^

The organism is protected against oxidative stress by the capacity of enzymes of the endogenous antioxidant system to act as powerful free radical suppressors. Among these enzymes are catalase (CAT), superoxide dismutase (SOD), and glutathione peroxidase (GPx).^24,25^ The latter is the main element of the antioxidant system involved in the response to chronic hyperglycemia, which deregulates H_2_O_2_ production. Since GPx catalyzes H_2_O_2_ into water by using glutathione (GSH) as a reducer, it is very active during the onset and development of diabetes.^25^

Unfortunately, the various animal models used to test possible therapies for hyperglycemia and diabetes all have serious shortcomings.^17–19,27^ Hence, there is an urgent need for an economical animal model capable of manifesting alterations in metabolic parameters very similar to those caused by the onset and progression of diabetes, especially type 2 diabetes, NODAT, and the respective complications. The creation of a reliable and low-cost model would not only facilitate the evaluation of new therapeutic strategies for type 2 diabetes, but also the search for the next generation of immunosuppressants that do not trigger the onset of diabetes.^16–19,27^

The aim of the current study was to examine the validity of a new, low-cost, non-obese model of type 2 diabetes engendered by administering a daily high dose of tacrolimus (an immunosuppressant) to Wistar rats for 4 weeks. The control rats were subjected to the same conditions, but without tacrolimus administration. The biochemical and antioxidant markers were evaluated two times: 1) on the last day of the week of animal acclimation to lab conditions (before treatment began, being the basal measurement and denominated week 0), and 2) after the 4-week tacrolimus treatment. This model generated a pathogenic condition that complies with the diagnostic criteria for type 2 diabetes established by the ADA.^6^ Furthermore, the mechanisms of pathogenesis appear to be similar to the ones occurring in the human disease.

## 2. Results

### 2.1. The fasting blood glucose level

**Figure 1.**
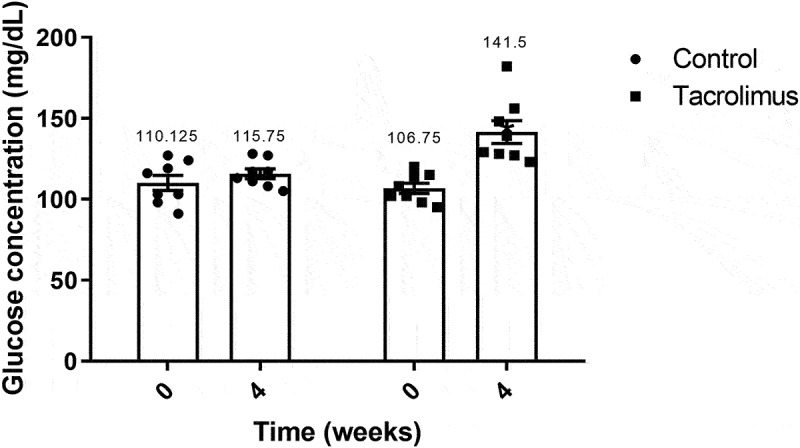
Fasting blood glucose concentration values at weeks 0 and 4. The graph of bars and points portrays a higher blood glucose concentration for the tacrolimus group at week 4 *versus* week 0, and higher at week 4 for the tacrolimus *versus* control group. There are two bars on the left and another two on the right, corresponding to the control and tacrolimus groups, respectively, in each case at weeks 0 and 4. Each bar represents the mean plus or minus the standard error, and each point expresses the average of three measurements made on each animal. The asterisk above the bar that corresponds to the tacrolimus group at week 4 indicates a significant difference compared to the tacrolimus group at week 0 and compared to the control group at week 4, with significance considered at 0.05 for the Student’s *t*-test. The control and tacrolimus groups each consisted of 8 animals, for a total of 16.

There was no significant difference in the level of blood glucose between the experimental and control groups at week 0 (basal), being in the range of 106.75–110.125 mg/dL. At week 4, however, a significant difference was indeed found between groups. The rats given tacrolimus had an average of 141.5 mg/dL and the control animals 115.75 mg/dL (Figure 1).

### 2.2. The glucose tolerance curve

**Figure 2.**
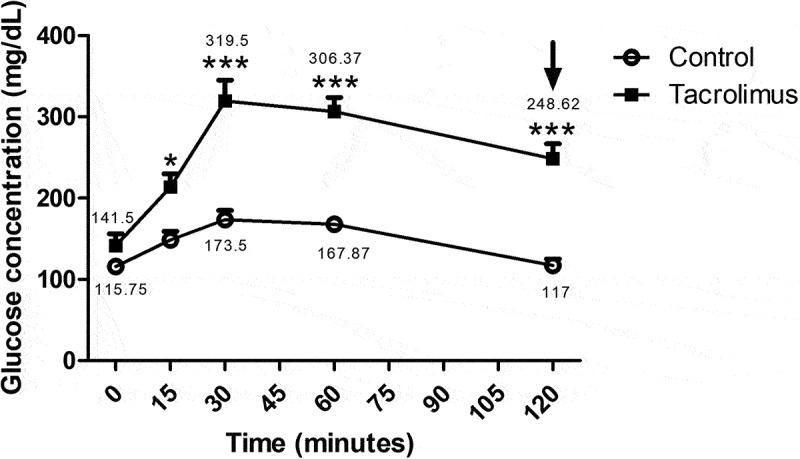
The glucose tolerance test, the animals were orally administered a dose of 1.5 grams of glucose per kilogram of body weight at week 4. The blood glucose concentration was measured at 0, 15, 30, 60 and 120 minutes, with three measurements taken at each time. The mean plus or minus the standard error of each measurement is represented as a point on the curve for each group. The asterisk located above the points of the upper curve indicates a significant difference between groups, with significance considered at 0.05 for the two-way repeated measures ANOVA test. The control and tacrolimus groups each consisted of 8 animals, for a total of 16.

From 0–120 min of the glucose tolerance test, the area under the curve was in the range of 141.5–248.625 mg/dL for the tacrolimus group and 115.75–117 mg/dL for the control at week 4. Following the administration of 1.5 g/kg of glucose (at week 4), a significant difference existed in the area under the curve between the two groups at 15 min, and a greater difference from 30–120 min (Figure 2).

### 2.3. The insulin tolerance test

**Figure 3.**
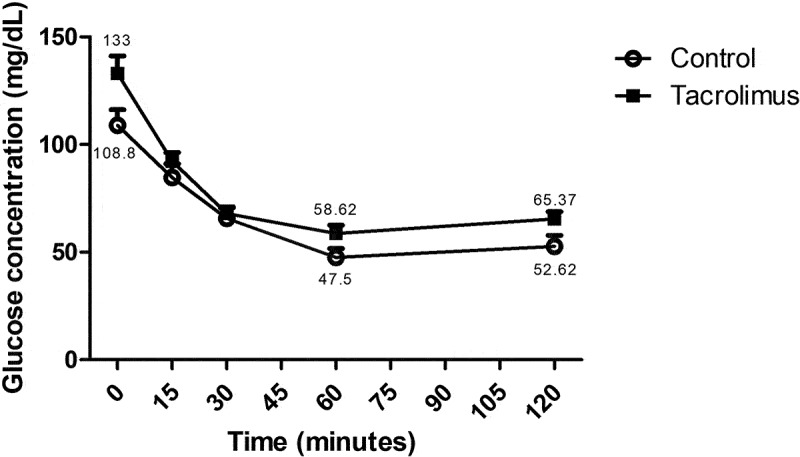
The insulin tolerance test, 0.5 international units of rapid-acting insulin per kilogram of body weight were administered intraperitoneally to each animal in the two groups at week 4. The blood glucose concentration was determined at 0, 15, 30, 60 and 120 minutes. Each point on the curve represents the mean plus or minus the standard error of three measurements. Significant differences, considered at 0.05, were analyzed with the two-way repeated measures ANOVA test. The curve of the tacrolimus group is slightly above that of the control group, indicating decreased insulin sensitivity. Each group consisted of 8 animals, for a total of 16.

The initial value of blood glucose before the insulin tolerance test (at week 4) was 108.875 mg/dL for the control and 133 mg/dL for the tacrolimus group. After injection of rapid-acting insulin, a steady decrease was found in the level of this hormone in both groups during 60 min until reaching 58.625 mg/dL and 47.5 mg/dL for the tacrolimus-treated and control animals, respectively. Subsequently, a continuous recovery was detected from 60–120 min, reaching 65.375 mg/dL and 52.625 mg/dL for the experimental and control groups, respectively (Figure 3).

### 2.4. The plasma insulin level

**Figure 4.**
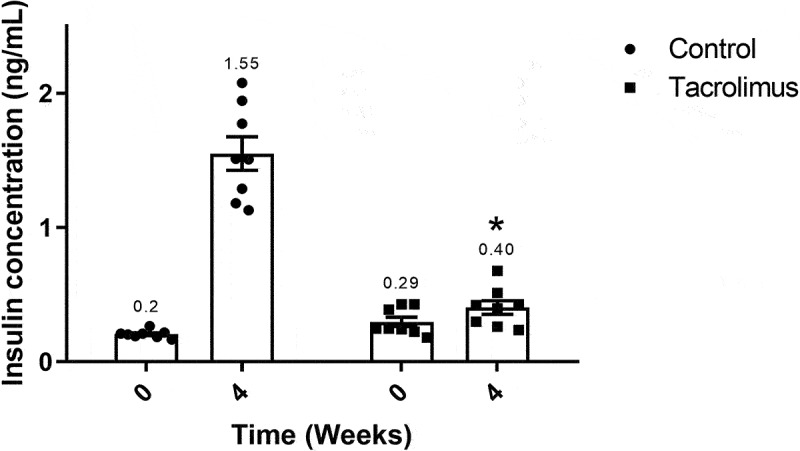
Plasma insulin concentration values at weeks 0 and 4. The graph of bars and points shows significantly greater values for the tacrolimus group at week 4 *versus* week 0, and greater values at week 4 for the tacrolimus *versus* control group. There are two bars on the left and two on the right, corresponding to the control and tacrolimus groups, respectively, in each case at weeks 0 and 4. Each bar represents the mean plus or minus the standard error, and each point expresses the average of three measurements made on each animal. The asterisk above the bar that corresponds to the tacrolimus group at week 4 indicates a significant difference with the control group, with significance considered at 0.05 for student’s *t*-test. Each group consisted of 8 animals, for a total of 16.

At week 4, the plasma insulin concentration in the tacrolimus group was in the range of 0.236–0.677 ng/mL, with an average of 0.404 ng/mL. The corresponding control value was 1.55 ng/mL, representing a significant difference (Figure 4).

### 2.5. The levels of total cholesterol and triglycerides

**Figure 5.**
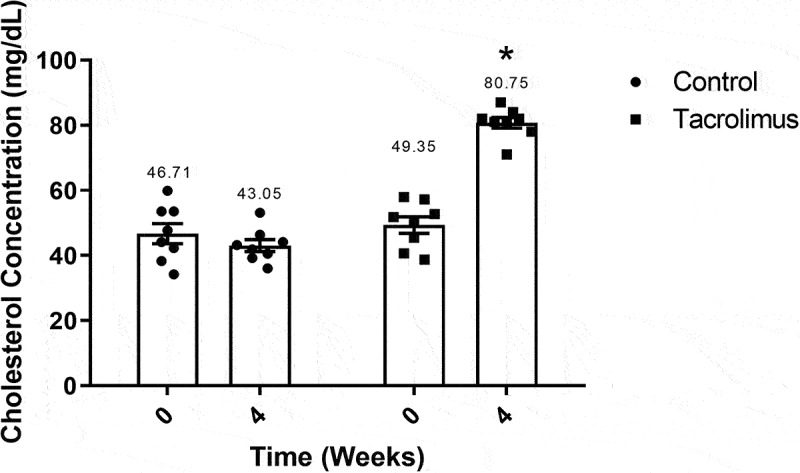
Total cholesterol concentration values are illustrated for weeks 0 and 4. The graph of bars and points portrays the higher level of cholesterol found in the tacrolimus group at week 4 *versus* week 0, and the higher level at week 4 for the tacrolimus *versus* control group. There are two bars on the left and two on the right, corresponding to the values for the control and tacrolimus groups, respectively, in each case at weeks 0 and 4. Each bar represents the mean plus or minus the standard error, and each point expresses the average of three measurements made on each animal. The asterisk above the bar that corresponds to the tacrolimus group at week 4 indicates a significant difference with the control group, with significance considered at 0.05 for the student’s *t*-test. Each group consisted of 8 animals, for a total of 16.

The total cholesterol of the two groups was within the normal range of 46.71–49.35 mg/dL at week 0. The experimental rats displayed an elevated average value of 80.75 mg/dL at week 4 compared to 49.355 mg/dL at week 0, representing a significant difference between measurement times. The week 4 value for the control animals was 43.05 mg/dL (Figure 5), indicating a significant difference between groups.

**Figure 6.**
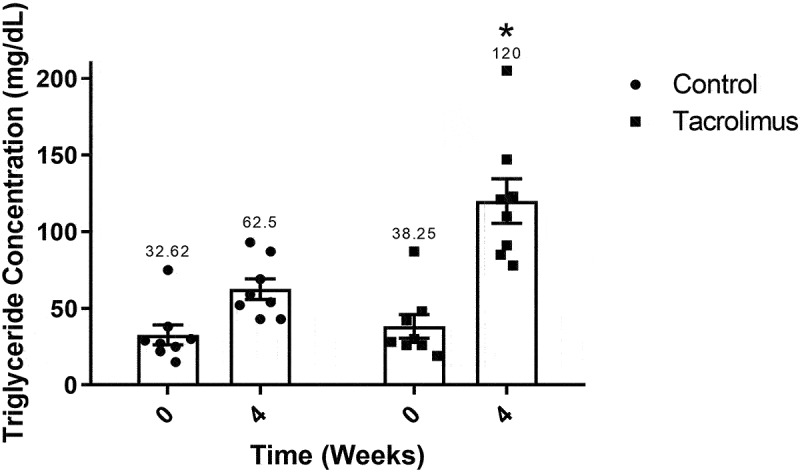
Triglyceride concentration values at weeks 0 and 4. The graph of bars and points displays a higher value for the tacrolimus group at week 4 *versus* week 0, and a higher value at week 4 for the tacrolimus *versus* control group. There are two bars on the left and two on the right, corresponding to the values for the control and tacrolimus groups, respectively, in each case at weeks 0 and 4. Each bar represents the mean plus or minus the standard error, and each point expresses the average of three measurements made on each animal. The asterisk above the bar that corresponds to the tacrolimus group at week 4 indicates a significant difference with the control group, with significance considered at 0.05 for the student’s *t*-test. Each group consisted of 8 animals, for a total of 16.

Triglycerides exhibited a similar behavior. Values for the control and tacrolimus-treated animals were 32 to 38 mg/dL at week 0, respectively. For the experimental group, a significantly higher mean concentration of triglycerides was found at week 4 (120 mg/dL) than week 0 (38 mg/dL). The level of triglycerides was significantly different between groups at week 4, being 120 mg/dL for the experimental group and 62.5 mg/dL for the control (Figure 6).

### 2.6. The activity of ALT, AST and GPx

**Figure 7.**
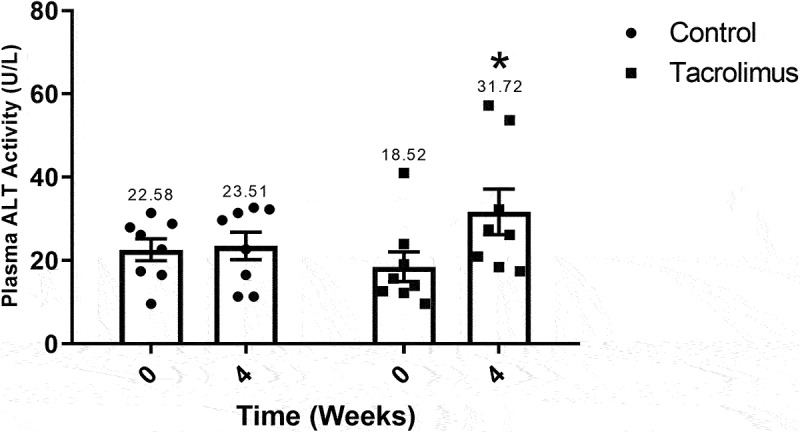
Alanine aminotransferase activity values at weeks 0 and 4. The graph of bars and points exhibits a higher value of ALT activity for the tacrolimus group at week 4 *versus* week 0, and a higher value at week 4 for the tacrolimus *versus* control group. There are two bars on the left and two on the right, corresponding to the control and tacrolimus groups, respectively, in each case at weeks 0 and 4. Each bar represents the mean plus or minus the standard error, and each point expresses the average of three measurements made on each animal. The asterisk above the bar that corresponds to the tacrolimus group at week 4 indicates a significant difference with the control group, with significance considered at 0.05 for the student’s *t*-test. Each group consisted of 8 animals, for a total of 16.

**Figure 8.**
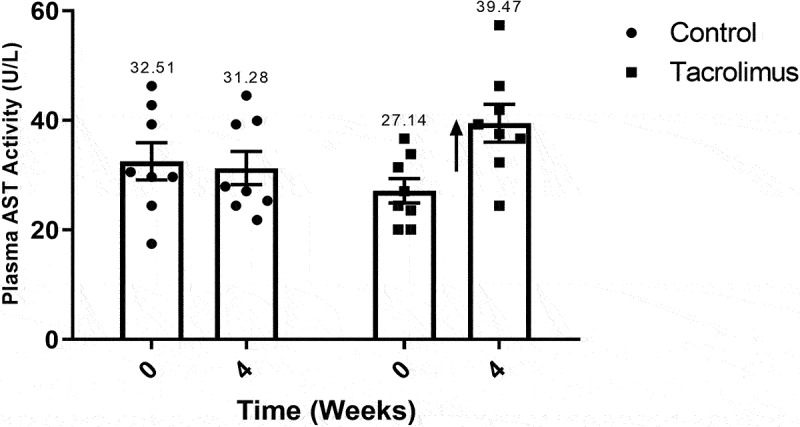
Aspartate aminotransferase activity values at weeks 0 and 4. The graph of bars and points depicts a greater value for the tacrolimus group at week 4 *versus* week 0, and a greater value at week 4 for the tacrolimus *versus* control group. There are two bars on the left and two on the right, corresponding to the control and tacrolimus groups, respectively, in each case at weeks 0 and 4. Each bar represents the mean plus or minus the standard error, and each point expresses the average of three measurements made on each animal. The vertical arrow pointing upwards, located at the side of the far right bar, indicates a tendency for enzymatic activity to weaken. Each group consisted of 8 animals, for a total of 16.

**Figure 9.**
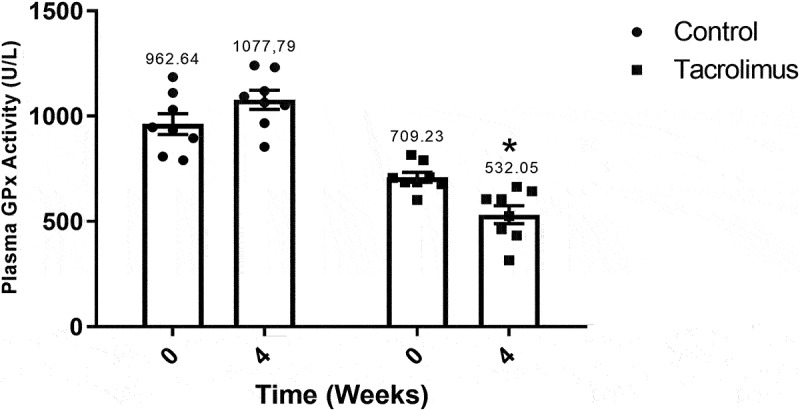
Glutathione peroxidase activity values at weeks 0 and 4. The graph of bars and points shows a greater value for the tacrolimus group at week 4 *versus* week 0, and a greater value at week 4 for the tacrolimus *versus* control group. There are two bars on the left and two on the right, corresponding to the control and tacrolimus groups, respectively, in each case at weeks 0 and 4. Each bar represents the mean plus or minus the standard error, and each point expresses the average of three measurements made on each animal. The asterisk above the bar that corresponds to the tacrolimus group at week 4 indicates a significant difference with the control group, with significance considered at 0.05 for the student’s *t*-test. Each group consisted of 8 animals, for a total of 16.

The activity of the ALT enzyme was significantly greater in the tacrolimus group at week 4 (31.7216 U/L) versus week 0 (18.5295 U/L). Contrarily, the average level in the control animals was practically unchanged during the study (Figure 7). The activity of AST increased from 27.1422 to 39.4734 U/L in the tacrolimus group during the 4-week treatment (Figure 8). GPx enzymatic activity was quantified by means of the glutathione (GSH)-coupled reaction, observing a significant difference in the tacrolimus-treated rats between week 0 (709.23 U/L) and week 4 (532.05 U/L). No difference was found between measurement times for the control (Figure 9).

### 2.7. Animal body weight

**Figure 10.**
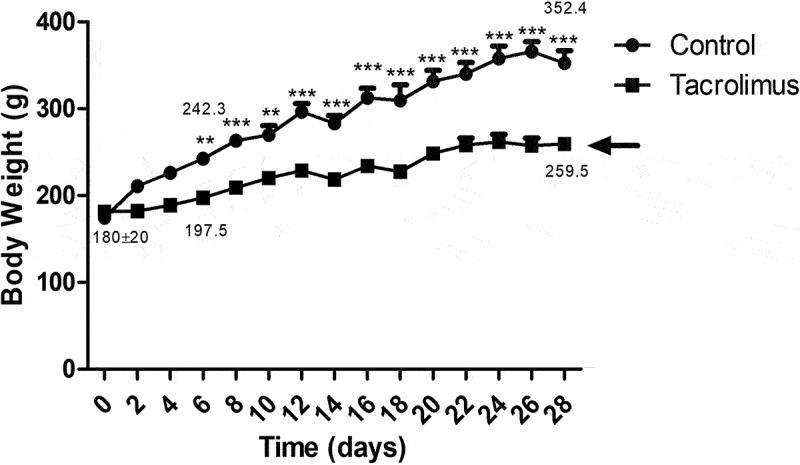
The body weight of the animals was assessed throughout the experiment and is depicted with two curves. The lower curve, representing less body weight, corresponds to the tacrolimus group. A difference between the curves can be appreciated immediately after the first administration of tacrolimus. Each point expresses the mean plus or minus the standard error. The asterisk placed on the values of the upper curve indicates a significant difference between the groups, with significance considered at 0.05 for the two-way repeated measures ANOVA test. Each group consisted of 8 animals, for a total of 16.

The basal weight of both groups of animals was 180 ± 20 g. A separation of the curves began after the first administration of tacrolimus and showed a significant difference as of day 6. At 4 weeks, the average weight was 259.525 g for the tacrolimus group and 352.462 g for the control (Figure 10).

### 2.8. The cell morphology of pancreatic tissue

**Figure 11.**
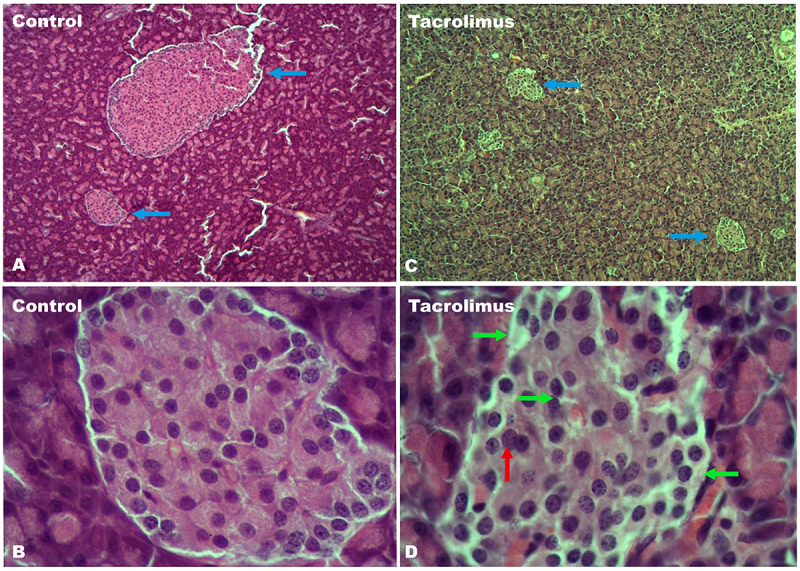
A view of the pancreatic tissue stained with hematoxylin-eosin is portrayed in four sections. Tissue samples were taken after the 4-week tacrolimus treatment. The coexistence of small and large islets in the control group is indicated by blue arrows in section A. In contrast, the sole presence of small islets in the tacrolimus rats is illustrated by blue arrows in section C. These two images were taken with 5x magnification. The structural integrity of normal pancreatic islets can be appreciated in the control tissue in section B. On the other hand, areas with a loss of structural integrity and of cell uniformity are seen in the tacrolimus-treated group, denoted by green arrows in section D. Additionally, there is an increase in connective tissue at the intercellular junctions of islet cells and between the islet cells and adjacent cells of the basement membrane. Finally, chromatin alterations are pointed out by red arrows. The latter two images were taken with 40x magnification.

At week 4, analysis of stained slices of pancreatic tissue from control rats revealed the coexistence of large islets and small islets (Figure 11A) with intact structural integrity (Figure 11B). In contrast, the tissue slices of the tacrolimus animals displayed only small islets (Figure 11C). These had undergone a loss of structural integrity (Figure 11D) secondary to an increase in connective tissue, found at the intercellular junctions of islet cells and between the islet cells and adjacent cells of the basement membrane. Although chromatin disturbances were observed, there was no evidence of apoptotic cells (characterized by a loss of contact with the rest of the cells as well as condensation of chromatin and fragmentation of the nucleus) or of infiltration by mononuclear immune cells.

## 3. Discussion

The current contribution describes a new, low-cost, non-obese model of type 2 diabetes capable of generating hyperglycemia and carbohydrate intolerance in rats after only 4 weeks of daily administration of tacrolimus (an immunosuppressive agent).^18,28^ For the proper modeling of type 2 diabetes, pancreatic β-cell dysfunction and/or insulin resistance must be induced. The tacrolimus-treated animals in this study exhibited the deterioration of pancreatic β cells and an insufficiency in the secretion of insulin, thus meeting the criteria proposed by the ADA for the diagnosis of type 2 diabetes and NODAT.^29^ The current model is low in cost, thus contrasting with expensive spontaneous models. To facilitate frequent testing of new therapies, a model of type 2 diabetes in rats should also rely on readily available materials, and easily produce and maintain a diabetic condition,^19,30^ which is the case for the present model.

At week 4, the fasting glucose level was significantly higher for the experimental than control group (141.5 vs 115.75 mg/dL, respectively). The Charles River® laboratories have reported similar data for their two principal type 2 diabetes animal models: a glucose level of ~150-200 mg/dL^31^ for 8-week-old Zucker rats (ZDF-Lepr^fa^/Crl)^32^ and **~**250 mg/dL for 10-week-old Gotto-Kakizaki (GK) rats.^33–36^

A model of type 2 diabetes should also be able to generate carbohydrate intolerance.^18,28^ In tacrolimus-treated rats, the level of carbohydrates at week 4 reached a peak concentration of 300 mg/dL at 30 min post-administration of 1.5 g of glucose and then decreased slightly to 248.625 mg/dL by 120 min. Hence, the curve is moved to the right, evidencing carbohydrate intolerance. This parameter reaches a value of 200–250 mg/dL in GK rats,^18,33,37^ manifesting a deterioration of carbohydrate homeostasis.^16^ Contrarily, Li *et al*. did not report such effects on glucose values. However, they concluded that oral administration of different doses of tacrolimus to Wistar rats causes changes in the structure and function of pancreatic β cells, closely related to glucose metabolism disorders.^38^

The elevated concentration of triacylglycerides is possibly due to the higher level of the very low-density lipoproteins (VLDL) that carry them. Through the effect of insulin, there is a decline in the inhibition of hormone-sensitive lipase in adipose tissue, thus increasing lipolysis and circulating levels of free fatty acids, the main substrate for the production of VLDL.^39–42^

Insulin resistance consists of a reduced sensitivity of cells to insulin molecules, which results from the compensatory increase in insulin secretion by pancreatic islets during chronic hyperglycemia (Figure 1A). This condition triggers a chain reaction of alterations. The first mechanism affected is a completely insulin-dependent pathway of glucose transport mediated by glucose transporter 4 (GLUT4), followed by insulin-induced phosphorylation of the tyrosine residues in insulin receptors 1, and finally the recruitment of the substrates necessary for glucose deposition in skeletal muscle cells.^43,44^ The alteration of the latter mechanism can be appreciated in the present study by the difference in the level of blood glucose in the insulin tolerance test at 120 min between the tacrolimus and control groups (65.375 mg/dL vs 52.625 mg/dL, respectively). Although the difference is not significant, the values suggest a decreased activation of insulin receptors 1 caused by an increased phosphorylation of tyrosine residues. In this sense, Pereira *et al*. noted that an increase in the phosphorylation of Ser and Thr leads to a decline in the activity of PI3K and Akt kinases and defects in the expression and function of GLUT4.^41,42,45^

The concentration of plasma insulin was herein quantified as a marker of the functional capacity of pancreatic β cells, finding a significant difference between the tacrolimus and control groups at week 4 (0.404 ng/mL vs 1.55 ng/mL, respectively), indicating hypoinsulinemia in the experimental animals.^18^ The same condition is also shown in 12-week-old GK rats, reported to have an average concentration of **~**1.25 ng/mL of insulin.^46^

Insulin secretion normally occurs when glucose enters β cells through GLUT2. The resulting increase in the ATP/ADP ratio causes K-ATP channels to close and the membrane to depolarize, triggering the opening of voltage-gated Ca^2+^ channels and consequently a greater level of intracellular Ca^2+^. The latter favors the fusion of the insulin-containing granules within the plasma membrane and their release into circulation.^13,21,22^

The low insulin levels in the experimental group suggest that the tacrolimus binding protein is highly concentrated in pancreatic β cells, as Tosur *et al*. reported.^47^ Tacrolimus is known to be a selective inhibitor of calcineurin, which is an essential protein for the proliferation of β cells. Hence, calcineurin/NFAT signaling could possibly be responsible for regulating β cell proliferation and the corresponding establishment of the cell mass capable of adequate insulin secretion.^8,48–50^

According to the aforementioned evidence, type 2 diabetes and NODAT seem to share the following phenomena: A) a diminished secretory capacity of β cells, B) a decrease in glucose-induced insulin secretion, C) an increase in the apoptosis of pancreatic islets, and D) the deterioration of the metabolic pathways involved in the synthesis of ATP and the nuclear factor of activated T-cells (NFAT). Since these mechanisms alter the consumption of mitochondrial oxygen, they reduce mitochondrial mass. Thus, it is likely that tacrolimus affects pancreatic β cells by damaging their cytoskeleton and decreasing membrane trafficking, which would diminish the production of ATP and the secretion of insulin.^10–12,51^

The greater activity of ALT and AST in the tacrolimus group can be directly attributed to the treatment. A previous study described the unaltered activity of these enzymes at low doses of tacrolimus and reduced insulin levels at higher doses.^52^ The reduction in insulin reveals a decline in β cell function (implying chronically elevated blood glucose) and has a substantial effect on the proper functioning of insulin-sensitive liver tissue, leading to altered glycogen metabolism and triglyceride storage and therefore chronically overactive hepatic enzymes.^22,53^

To explore the metabolism of lipids after tacrolimus treatment, the determination of total cholesterol and triglycerides was carried out at weeks 0 and 4. A tendency to hypercholesterolemia was indicated by the almost two-fold boost in the mean concentration of total cholesterol in the tacrolimus versus control group at week 4 (80.75 mg/dL vs 43.05 mg/dL, respectively). The control value was within the normal parameters (44 mg/dL) for rats under 16 weeks of age.^54^ The same pattern was observed with the mean concentration of triglycerides for the tacrolimus and control groups at week 4, finding 120 mg/dL versus 62 mg/dL, respectively. The high level for the tacrolimus-treated rats is very close to the average level of triglycerides (100 mg/dL) attributed to GK rats.^55^ Thus, the current values of total cholesterol and triglycerides, very similar to those described for the GK model,^56^ evidence the alteration of lipid metabolism.

Regarding GPx activity at week 4, the tacrolimus group displayed a lower level (532.059 U/L) than the control (709.237 U/L). Although GPx is highly effective as an endogenous antioxidant in relation to many ROS, it apparently does not counteract hydroperoxides. According to reports in the literature, tacrolimus treatment generates an elevated expression of ROS, including H_2_O_2_, and the latter probably leads to mitochondrial dysfunction.^57^

Tacrolimus-induced hyperglycemia, hyperlipidemia and oxidative stress in the experimental group triggered changes in the architecture of pancreatic islets, causing a deterioration of the functional cells responsible for synthesizing and secreting molecules involved in regulating glucose homeostasis. These alterations closely resemble those described in the morphological analysis of the endocrine pancreas of GK rats. Additionally, there was no inflammatory cell infiltrate, an element found in some animal models of type 1 diabetes,^30,58–60^ and the monitoring of animal body weight demonstrated the non-obese nature of the present model (a feature it shares with the GK model).^61^

## 4. Conclusion

The current contribution evaluates a new model of type 2 diabetes based on exposing rats to a daily high dose of tacrolimus for 4 weeks. The resulting alterations in metabolic parameters are probably engendered by the deterioration of pancreatic β cell function, suggesting a pathophysiological mechanism similar to type 2 diabetes. The complete pathogenesis of this disease is expressed in the new model (as in the GK model): dysfunctional pancreatic β cells, hyperglycemia, insulin resistance, carbohydrate intolerance, oxidative stress, and elevated levels of cholesterol and triacylglycerides. Unlike complex (e.g., spontaneous) models, the tacrolimus model is not costly. In contrast to the models based on diets rich in carbohydrates and lipids, the present model is non-obese and easily developed in a short period of time. Moreover, the mortality rate is null. Hence, this new model should certainly facilitate the testing of new therapies aimed at curing or stopping the progression of diabetes, and the search for the next generation of immunosuppressants that do not trigger the onset of type 2 diabetes or NODAT.

## 5. Material and methods

### 5.1. Animal care

Sixteen 8-week-old male Wistar rats (150 ± 20 g) were provided by the Bioterium of the Universidad Autónoma Metropolitana (UAM). All animals were given water and food (Rat Chow 5012, Purina®) *ad libitum*, except when fasted for 8 hours prior to sampling and testing. The procedures performed on the animals comply with the requirements of the Internal Committee for the Care and Use of Laboratory Animals of the Escuela Superior de Medicina (ICCULA-01/27-09-2018), the “*Technical specifications for the production, care and use of laboratory animals*” published by the Secretary of Agriculture in Mexico (SAGARPA, NOM-062-ZOO-1999), and “*The guide for the care and use of laboratory animals*” of the National Research Council.

The rats were allowed 1 week to adapt to an isolated room with a directional airflow before being separated into two groups. On the last day of the adaptation period (week 0), the basal values of biochemical and antioxidant markers were measured. The experimental group received a daily high dose (1 mg/Kg bw) of tacrolimus (Sigma-Aldrich, 1642802–150 MG) injected subcutaneously, as reported previously. The untreated control was subjected to the same hygienic and dietary conditions. At the end of the 4-week treatment, blood samples were taken again to measure the biochemical and antioxidant markers. Additionally, tissue samples were extracted.

### 5.2. Collection of the blood sample

At weeks 0 and 4, the animals were anesthetized with sodium pentobarbital (20 mg/Kg of bw) to extract blood by puncturing the left lateral vein of the tail.

### 5.3. TheGlucose tolerance test

At week 4, all the rats were fasted for 8 hours and then orally administered 1.5 g of glucose through a gastric cannula. Subsequently, the blood glucose concentration was measured with an Accu-Chek® Performa glucometer (Roche) at 0, 15, 30, 60 and 120 min.

### 5.4. The insulin tolerance test

At week 4, all the animals were fasted for 8 hours and interperitoneally injected with 0.5 IU/Kg of rapid-acting insulin (Insulex® R, PISA). The blood glucose concentration was then quantified by means of an Accu-Chek® Performa glucometer (by puncturing the right lateral caudal vein) at 0, 15, 30, 60 and 120 min.

### 5.5. Biochemical parameters

At weeks 0 and 4, determinations were made of the glucose concentration and other blood parameters. Cholesterol was evaluated by the enzymatic end-point method and the cholesterol (CH200) kit (Randox, UK), triacylglycerides by the GPO-PAP method and the triglycerides (TR210) kit (Randox, UK), and plasma insulin with the rat/mouse insulin ELISA (635151) kit (Merck-Millipore, MO, USA). Quantitative *in vitro* analysis of the activity of ALT and AST was carried out by using the standardized and optimized UV method and the ALT (AL1200) and AST (AS1202) kits (Randox, UK), in accordance with the guidelines of the International Federation of Clinical Chemistry (IFCC). The activity of glutathione peroxidase (GPx) was assessed with the UV method and the Ransel (RS504) kit (Randox, UK).

### 5.6. The cell morphology of pancreatic tissue

Upon completing the glucose tolerance and insulin tolerance tests and taking blood samples at week 4, the animals were deeply anesthetized with 60 mg/Kg of sodium pentobarbital to comply with the humane endpoint criterion. Gravity exsanguination was performed with a 4% formaldehyde infusion through a catheter inserted into the left ventricle. A cut was made at the level of the right appendage to allow for the complete fixation of all organs and the extraction of pancreatic tissue samples. Subsequently, the samples were embedded in paraffin blocks to be serially sliced into 4 µm sections, which were mounted on slides and subjected to conventional hematoxylin and eosin (H&E) staining for observation by light microscopy.

### 5.7. Statistical analysis

Data were examined on Sigma-Plot software (SYSTAT, USA). The values are expressed as the mean ± standard error and statistical significance was considered at p < .05.

## Supplementary Material

Supplemental Material

## Data Availability

The data that support the findings of this study are available from the corresponding author upon reasonable request.

## Highlights

1. Tacrolimus generates a diabetes-like condition in rats in 4 weeks.
2. The new low-cost model produces the complete pathophysiology of the disease.
3. The molecular mechanisms are probably based on the progressive loss of β-cell function.
