# Supplementary material for: Modeling type 2 diabetes in rats by administering tacrolimus
Source: Islets. 2022 Mar 29;14(1):114–27. doi: 10.1080/19382014.2022.2051991 (PMC8966987; doi:10.1080/19382014.2022.2051991)
Supplement: Supplemental Material [file KISL_A_2051991_SM7085.docx]

**Supplementary material**

**1. Material and methods**

**1.1. Animal Care**. Sixteen 8-week-old male Wistar rats (150 ± 20 g) were provided by the Bioterium of the Universidad Autónoma Metropolitana (UAM). All animals were given water and food (Rat Chow 5012, Purina®) *ad libitum*, except when fasted for 8 hours prior to sampling and testing. The procedures performed on the animals comply with the requirements of the Internal Committee for the Care and Use of Laboratory Animals of the Escuela Superior de Medicina (ICCULA-01/27-09-2018), the “*Technical specifications for the production, care and use of laboratory animals*” published by the Secretary of Agriculture in Mexico **(**SAGARPA, NOM-062-ZOO-1999), and “*The guide for the care and use of laboratory animals*” of the National Research Council.

The rats were allowed 1 week to adapt to an isolated room with a directional airflow before being separated into groups. On the last day of the adaptation period (week 0), the basal values of biochemical and antioxidant markers were measured. The experimental group received a daily high dose (1 mg/Kg bw) of tacrolimus (Sigma-Aldrich, 1642802-150MG) injected subcutaneously, as reported previously. The untreated control was subjected to the same hygienic and dietary conditions.

**1.2. The glucose tolerance test**. At week 0, all the rats were fasted for 8 hours and then orally administered 1.5 g of glucose through a gastric cannula. Subsequently, the blood glucose concentration was measured with an Accu-Chek® Performa glucometer (Roche) at 0, 15, 30, 60 and 120 min.

**1.3. The insulin tolerance test**. At week 0, all the animals were fasted for 8 hours and interperitoneally injected with 0.5 IU/Kg of rapid-acting insulin (Insulex^®^ R, PISA). The blood glucose concentration was then quantified by means of an Accu-Chek^®^ Performa glucometer (by puncturing the right lateral caudal vein) at 0, 15, 30, 60 and 120 min.

**2. Results**

**2.1. The glucose tolerance curve**

From 0-120 min of the glucose tolerance test, the area under the curve was in the range of 106.75-104.125 mg/dL for the tacrolimus group and 110.125-103.375 mg/dL for the control at week 0. Following the administration of 1.5 g/kg of glucose (at week 0), a significant difference existed in the area under the curve between the two groups at 15 min, and a greater difference from 30-120 min (Fig. 1).

**Figure 1. The glucose tolerance test.** A dose of 1.5 g/kg of glucose was orally administered to the animals at week 0 (n=8 and N=16). The blood glucose concentration was then measured at 0, 15, 30, 60 and 120 min. Data are expressed as the mean ± SEM (two-way RM ANOVA).

**2.2. The insulin tolerance test**

The initial value of blood glucose before the insulin tolerance test (at week 0) was 110.25 mg/dL for the control and 98.37 mg/dL for the tacrolimus group. After injection of rapid-acting insulin, a steady decrease was found in the level of this hormone in both groups during 60 min until reaching 47.75 mg/dL and 50.25 mg/dL for the tacrolimus-treated and control animals, respectively. Subsequently, a continuous recovery was detected from 60-120 min, reaching 40.62 mg/dL and 47.37 mg/dL for the experimental and control groups, respectively (Fig. 2).


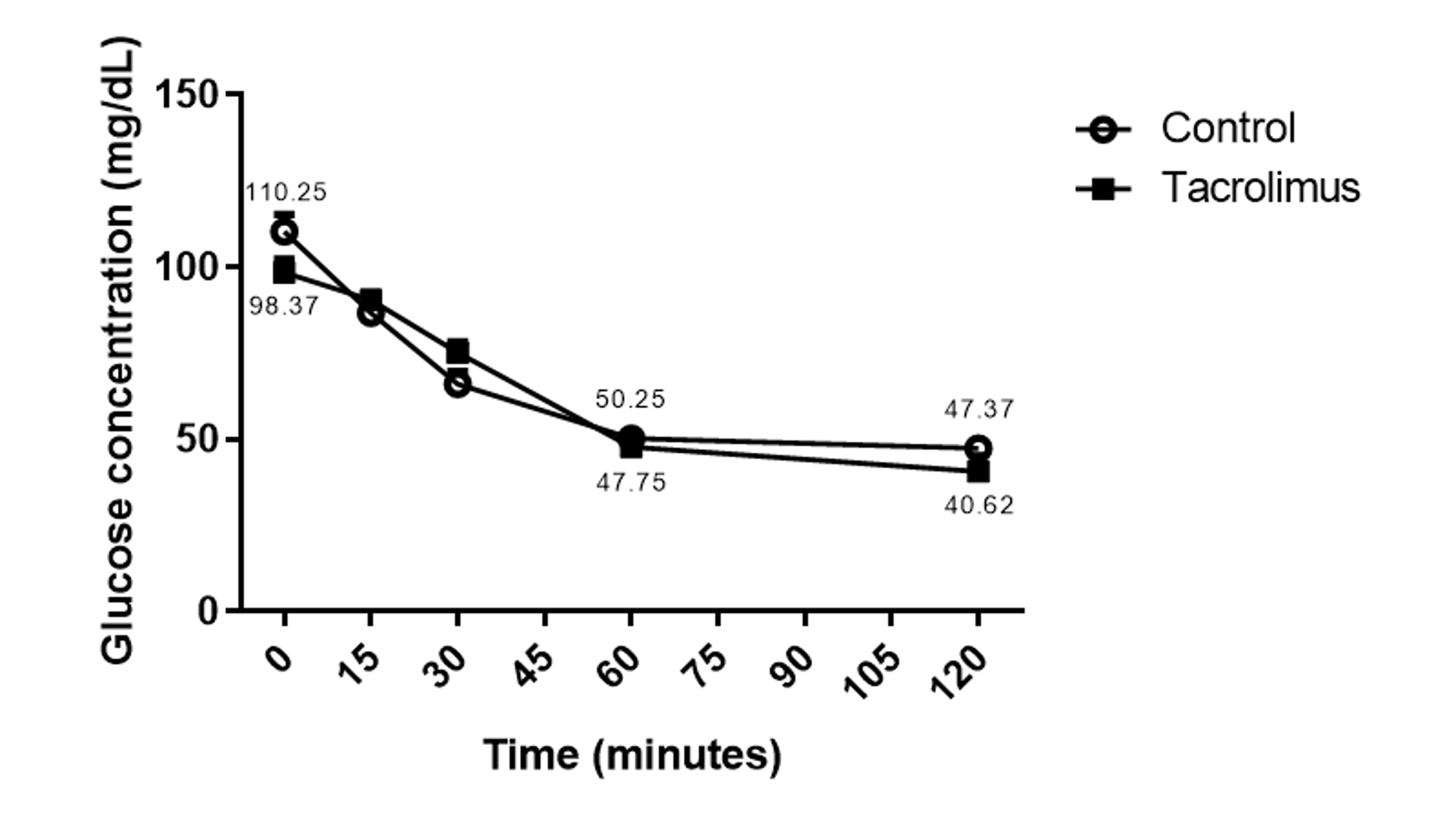


**Figure 2. The insulin tolerance test.** At week 0, 0.5 IU/Kg of rapid-acting insulin was intraperitoneally administered to both groups. Subsequently, the blood glucose concentration was determined at 0, 15, 30, 60 and 120 min (n=8 and N=16). Data are expressed as the mean ± SEM (two-way RM ANOVA).
